# The Alpha Hypothesis: Did Lateralized Cattle–Human Interactions Change the Script for Western Culture?

**DOI:** 10.3390/ani9090638

**Published:** 2019-08-31

**Authors:** Andrew Robins

**Affiliations:** Centre for Animal Welfare and Ethics, School of Veterinary Science, University of Queensland, Gatton Campus, Gatton, Queensland 4343, Australia; arobins@operamail.com

**Keywords:** cattle, lateralization, domestication, welfare, ethology, interspecies, communication, directional asymmetry, symbolism, ancient religion

## Abstract

**Simple Summary:**

The domestication of cattle was a key innovation early in the development of Western civilization. Cattle provided the main tractor force to enable broad-scale agriculture and the land transportation of goods. Their initial significance was religiously celebrated as the bull-like creator god *’El*, in Canaan (modern day Lebanon and Syria), and for over 6000 years in Ancient Egypt as the sky-goddess *Hathor*, often depicted as a sacred cow. In addition, the Ancient Egyptian hieroglyphic logogram of a horned ox head profile viewed from either the left or right side was used to signify the concept of “wisdom”. Stylizations of the ox head logogram seen from the left, and not right side have recently been found used in Egyptian graffito dating from around 1900 BCE (Before the Common Era). The strings of symbols have provided the earliest known examples of writing using phonemes such as used in the modern Western alphabet. The use of the directionally-asymmetrical left side ox-head symbol to represent a specific phoneme subsequently migrated around the Eastern basin of the Mediterranean Sea variously as ’al, ’el, alep, allup, eleph and alif in Ancient Semitic cultures, including Phoenician, Hebrew and Arabic, before becoming “alpha” in Ancient Greece by 700 BCE. For reasons that have not been fully understood, the ox-head symbol has always been positioned as the first letter in the lexicographical order of the respective Western languages. This review outlines the etymology of the strongly conserved position and directional asymmetry of the alpha symbol, and of its religious connection. In the light of recent behavioural studies, the hypothesis was presented to argue that the directional asymmetry of alpha represents early recognition and critical importance of behavioural lateralization in domesticated cattle when interacting with their human handlers.

**Abstract:**

Domestic cattle possess lateralized cognitive processing of human handlers. This has been recently demonstrated in the preference for large groups of cattle to view a human closely within the predominantly left visual field. By contrast, the same stimulus viewed predominantly within the right visual field promotes a significantly greater frequency of dispersal from a standing position, including flight responses. The respective sets of behaviours correspond with the traditional terms of “near side” for the left side of cattle and horses, and the “off” or “far side” for the right side. These traditional terms of over 300 years usage in the literature communicate functional practicalities for handling livestock and the recognition of lateralized cognitive processing. In this review, the possibility of even earlier recognition and the significance of laterality in cattle-human interaction was argued, from the earliest representations of the letter "A", originally illustrated from nearly 4000 years before the present time as the head of an ox as viewed not from the front or from the right, but from the left (near) side. By extension, this knowledge of lateralization in cattle may represent the earliest written example of applied ethology—the study of the behaviour of animals under human management.

## 1. Introduction 

The aim of this paper was to present an overview of recent findings of lateralized cognitive processing in domestic cattle for human-cattle interactions and raise the possibility that the nature of this interspecies relationship has been known for at least 4000 years. Specifically, that lateralized preferences of early domesticated cattle were symbolized as the left side of the head of an ox—which became the first letter of the modern Western alphabet, “A a”. Current understanding of the etymology of the modern phonetic alphabet includes the earliest-known appearance of about 1900 BCE, within the area of Thebes and Luxor in the Upper Nile. Moreover, the system of phonemes probably arose from an alliance of Ancient Egyptian and Canaanite cultures, potentially related to the military functions required during that period. Most symbolic elements of that early alphabet remain conserved throughout the modern Western world as majuscule (capital) letters. This conservation resisted cultural changes not only in the direction of written text, but also in the reuse of some symbols for new sounds—as has occurred for the modern letter “A” for one of five vowel sounds. The “A” symbol was originally stylized as the horned head of an ox. For over one thousand years, this symbol was portrayed with the ox head almost invariably viewed from the left side, before clockwise rotation to its current uprighted position was formalized by the Ancient Greeks and Romans. The uprighted symbol was used exclusively for nearly one thousand years before the development of cursive text symbols that recalled the ancient convention of left-sided asymmetry that we see today as the minuscule (lower case) letter “a”. The significance of the directional asymmetry of the ox head alpha has not been previously considered in the scientific literature. 

## 2. Comparative Vertebrate Lateralization

“Lateralization” is a highly conserved feature of extant vertebrate species, referring to specialized neural and cognitive processing carried out predominantly within either the right or the left side of the brain (e.g., [1,2]). The lateralized organization avoids duplication of functions carried out on both sides of the brain, and clusters related functions together to optimize processing speed while reducing the energy requirements of neural processing (e.g., [2,3]). Brain lateralization may well be an example of evolutionary fitness-maximization, as invertebrates are also increasingly reported with lateralized nervous systems whilst lacking a true brain [4]. Within vertebrate species, the strength of lateralization corresponds to a conferred advantage to cognitive processing speed, and hence, survivability, when responding to environmental cues such as a physical or predatory threat. 

**Figure 1:**
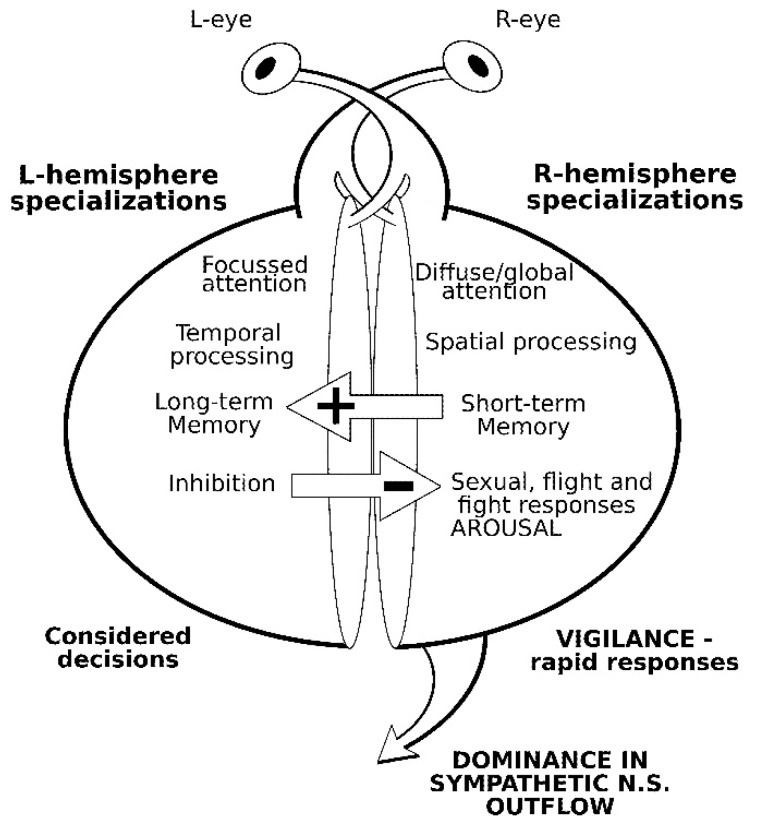
Summary of some cognitive functions found specialized within the left and right hemispheres of the vertebrate brain. Specialized functions listed for each side of the vertebrate brain are concluded from lateralized visual responses reported in at least two vertebrate classes (i.e., fish, amphibians, reptiles, birds, and mammals: [6]). The pathways from the eyes are illustrated schematically to indicate the dominance of visual processing carried out by the respective hemisphere —in many vertebrates, both ipsilateral and contralateral optic fibre projections exist, however, the contralateral pathways are found to dominate initial processing. In cattle and in horses the contralateral projections comprise 80%–87% of the visual pathways, by comparison to 50% found in humans, reflecting the general vertebrate pattern that species with laterally placed eyes have far more contralateral than ipsilateral projections [11]. The interhemispheric transfer of processing for short- to long-term memories shown in studies in which novel and potentially threatening stimuli are first attended to preferentially using the left eye before preferentially viewing the familiar stimulus using the right eye thereafter (e.g., chicks: [12]; lizards: [13]; toads: [14]; cattle: [15]). This is illustrated schematically as a positive arrow. The interhemispheric inhibition of right-hemisphere reactive responses to various visual stimuli by left-hemisphere specializations is illustrated as a negative arrow (see text for examples). The autonomic nervous system is also found to be under lateralized control. Here is highlighted the dominance of the right side of the brain for control of the sympathetic nervous system—primarily concerned with the physiological and behavioural responses of flight and fight [16,17]. By contrast, the parasympathetic nervous system control has not been conclusively shown to be preferentially controlled by either side of the vertebrate brain [16].

Figure 1 highlights a range of specialized cognitive functions of visual processing that have consistently been found to be specialized respectively within the two halves of the brain across multiple vertebrate species, at the population level. Studies from at least two vertebrate families (i.e., fish, amphibians, reptiles, birds and mammals) were included to present the current understanding of lateralized visual processing. The illustrated summary is a simplification of findings to illustrate patterns of visual processing—as judged by the behavioural and physiological responses that are evoked by specific visual stimuli. The responses may vary between species in degree or strength of lateralization in various contexts, probably depending on local ecological adaptations (e.g., [5]). Specific motor responses—such as handedness or limb preferences in response to a given visual target—were not addressed in this review. 

In broad terms, cognitive specializations of the right hemisphere deal primarily with responses to real-time, concrete cues from the immediate environment in a ‘bottom-up’ processing arrangement. These include vigilance functions (e.g., [6]; Figure 1). Analytical visual processes carried out in the right side of the brain are chiefly concerned with detecting change to the visual world with regard to specific and fine details. Importantly, the right side of the brain is asymmetrically connected to the sympathetic nervous system responsible for directing “flight and fight” responses, and also sexual responses, where together rapid increases in the physiological state are required for survival of the individual, and viability of the species. Noteworthily, a recent series of studies of a diverse range of mammalian taxa found that the infant preferentially monitors its mother within the left visual field during slow travelling, indicating the presence of a right hemisphere specialization for socio-emotional processing possibly associated with that hemisphere’s more general specialization for visuospatial processing [7]. By contrast, lateralized preferences for mothers viewing their infants were generally non-significant in routine non-threatening situations, but were found to emerge as a significant left eye/right hemisphere bias in stressful and potentially threatening contexts [7]. Similar lateralized relationships in calf-cow pairs of domestic cattle have been recorded but not yet published [8].

By contrast to right hemisphere specializations, cognitive specialized processes of the left hemisphere are characteristically referred to as considered, or ‘top-down’ processing (reviewed in [6]). Interactions between the two hemispheres illustrate the relative significance of these complementary but contrasting modes of analysis. Examples of left-brain lateralized processing involve recalled or abstract cues and stored templates of previous responses, such that individuals may be able to predict future outcomes using simple cause-and-effect relationships. Figure 1 illustrates this dominance of the left hemisphere for inhibiting right hemisphere responses associated with stress or arousal (see [6]). Recent evidence of top-down modulation of visually-evoked responses in the thalamus of pigeons supports the general vertebrate model presented in Figure 1, indicating a superiority of the left hemisphere to discriminate and categorize stimuli that the birds had had prior visual experience (i.e., long term memory) while biasing the right hemisphere towards responses to the immediate environment [9]. Furthermore, in experimental trials designed to place both hemispheres in conflict when responding to visual stimuli that were previously learned by either left or right eye systems, the left hemisphere of pigeons is able to dominate the right hemisphere by choosing the stimulus that was previously learned with the right eye [9].

The characteristics of top-down processing specialized in the left and not right hemisphere of vertebrates are also supported by findings of experience-dependent processes of the left hemisphere inhibiting or attenuating right hemisphere responses to fearful visual stimuli (reviewed in [6]). In zebrafish, the left and not right dorsal habenula is found responsible for attenuating the fear response of freezing after the experience of an unexpected negative experience—an electric shock [10]. 

The process of habituation or familiarity to a novel event is an example of experience-related changes in hemispheric dominance that is carried out differentially on either side of the brain, and this is illustrated schematically as a positive arrow in Figure 1 (reviewed in [6]). In other words, the right side of the brain is predominantly used first to assess the likelihood of a threat or value of a given novel stimulus, before the left side of the brain compares the stimulus with known or likely outcomes (reviewed in [6]). In some regards, the known processing modes of the respective hemispheres appear to correspond with this right-to-left directional transfer. Specifically, the right hemisphere is concerned with early detection of changes in the environment and finer details of stimuli, with the left hemisphere concerned with broad categorizations and abstracted relationships with past events (i.e., temporal processing)—functions that are consistent with long-term memory storage and consolidation (reviewed in [6]). First clearly demonstrated in domestic chicks, the right-to-left directional interhemispheric transfer of specific and detailed visual cues into long-term memory storage has been demonstrated in a range of vertebrates tested individually (e.g., imprinting memories in domestic chicks: [18,19]; discriminated avoidance learning in chicks: [12]; toads responding to unfamiliar prey: [14]), and in groups (i.e., lizards learning to feed on unfamiliar prey items: [13]). The first report of such interhemispheric visual processing in mammals involved rapid familiarization to novel challenging stimuli presented to domestic cattle tested in groups of up to 33 individuals [15], and is discussed in greater detail below.

### 2.1. Social Facilitation Hypothesis and Domestication

Individuals within a given species may vary in the degree of lateralization they express in motor and perceptual or cognitive processing compared with a population-level laterality. When comparing species population-level lateralization, strong and significant associations have been found between the directionality of behaviour and the degree of socialization that species exhibits [2,20]. Thus, the pressures of rapid coordination within a massed group to avoid collision whilst evading predation present simultaneous and competing cognitive processes that are best carried out simultaneously on either side of the brain [2]. The benefit conferred by lateralized, social consistency of behaviour and the processing efficiency it provides is presumably outweighed by the cost of any predictability in response that a given predator may be able to exploit, and it is likely that population-level asymmetries are socially facilitated [3,21].

The development of an ‘‘evolutionarily stable strategy’’ model reflecting the twin pressures of social cohesion and predator exploitation has been formulated, showing asymptotic and mirror-imaged trends to stability in the lateralized and complementary modes of analysis possessed by prey and predators [2,3,22]. It is arguable that while such an evolutionarily stable strategy model of lateralization may have value for single prey—single predator species interactions—it is a potentially more useful model for explaining lateralization within the artificial selection process of domestication [15]. Indeed, domestication might well be a fitness-maximizing process of selection for lateralization of responses—principally social conformity and herding tendency—and rapid adaptation to handling stressors, particularly object categorization and learning of positive associations. The desirable attributes of a domestic animal responding to a human handler (short flight distance; low reactivity to humans or sudden changes in environment; readily habituated; may approach and solicit attention from the human: [23]), coincide well with the list of higher cognitive functions found to be lateralized in the general vertebrate model presented in Figure 1 [6]. Animals selected for docility may well be lateralized for efficient and appropriate processing of stimuli related to intensive management. By contrast, individuals prone to comparatively unpredictable and therefore dangerous behaviour are rapidly culled from the breeding stock—in the same way that outliers in a natural herd setting are more likely to fall prey. A more recent expansion of the evolutionarily stable strategy model postulated that in the absence of predatory or competitive factors, population-level lateralization can be maintained internally—within the group—by socially-cooperative interactions [24,25]. The expanded model is probably also consistent with the socially-external process of artificial selection, stabilizing and promoting population-level lateralization wherever production values are enhanced. Indeed, Darwin considered the effect of domestication to be a significant selection pressure, devoting the first chapter of “Origin of the Species” and two subsequent volumes “On the Variations of Plants and Animals under Domestication” to the phenomenon [26,27]. 

It is important to note that different aspects of a given social environment are found to be under lateralized processing. Left eye/right hemisphere processes are found chiefly as awareness of where the social group members are located moment-to-moment, with regard to that hemisphere’s attention to visuospatial relationships and broad attention to cues indicating a potential threat such as a predator or social competitor (e.g., [6]). Right eye/left hemisphere processes appear to be more concerned with knowing or inferring where specific individuals are positioned within an established social hierarchy (e.g., [28]). These attributes are probably associated with the left hemisphere’s ability to distinguish individual facial characteristics based on fine feature details, in addition to abstracted matching with stored recollections.

### 2.2. Lateralized Visual Processing in Domestic Cattle

There are comparatively few studies of lateralized visual behaviour in cattle, and published findings have both corresponded to and contrasted with the general vertebrate pattern of brain lateralization (see Figure 1). Of the latter category, the tendency for both domestic cattle and horses to prefer to have humans positioned closely within the immediate left visual field—the “near side”—appears paradoxical, as this is the side specialized for responding to changes in the immediate environment, such as threats from predators and social competitors. As discussed in detail below, an explanation for this apparent paradox in preferential viewing was developed from experimental findings in successive studies and collectively summarized in Table 1 to highlight the important role of interhemispheric processing outlined earlier in Figure 1.

The first report of lateralized visual processing in cattle involved a simple challenge experiment of groups of between 17 and 33 cattle tested for viewing preferences in responding to an unfamiliar experimenter walking through the centre of the herd [15]. In successive trials, the experimenter additionally wore a succession of potentially threatening and novel apparatus (i.e., the opening and closing of umbrella, a long Y-shaped pole, and an idling 2-stroke engine). In each first trial where the test stimuli were novel, a significant left-eye preference was found for monitoring the approach of an unfamiliar human with and without additional apparatus (Table 1: [15]). However, on the second exposure during the return approach less than 10 minutes later, the viewing preferences of the cattle switched to a weak but significant right eye preference to maintain the respective stimuli within the right visual field, indicating rapid habituation to each test stimulus [15]. Thus, the trials demonstrated that cattle possessed both a left eye/right hemisphere lateralization for attending to novel stimuli and a subsequent reversal to right eye/left hemisphere preference to indicate a rapid process of familiarization. 

A follow-up study of visual challenge responses utilized large numbers of cattle in a commercial feedlot (90–200 cattle in each group, 14 or 18 groups tested in two challenge tests with replicates: [29]). In the first series of experiments where the centre of each group of cattle feeding along a bunk was repeatedly approached by a novel experimenter, cattle departed the bunk significantly less frequently whilst viewing the experimenter within the immediate left visual field—the “near” side. By comparison, there were a greater variety of behavioural responses, including flight responses, observed in cattle viewing the approach of the experimenter predominantly within their right visual field [29]. The authors speculated that the small cohort of cattle taking flight when viewing the novel experimenter within the right—the “off” side—and not the left visual field, as presented in the general vertebrate pattern (cf. Figure 1), did so due to cognitive dissonance [29]. Therefore, when presented with such novel unfamiliar stimuli that the preferred or specialized visual system for analyzing such information could not be easily utilized, a small but significant number of cattle chose to take flight to avoid the conflict in processing demands [29].

In a second series of challenge tests using the same feedlot cattle groups, an experimenter wore a nuisance mask over the nose and mouth to create a novel stimulus, and walked alongside the feed bunk without otherwise interacting with the feeding cattle. Here, a different set of lateralized visual responses were observed in contrast to the first series of challenge tests [29]. The cattle were significantly more likely to leave the feed bunk while viewing the approach of the masked experimenter within the left visual field, while remaining comparatively undisturbed from their feeding in response to the same stimulus viewed approaching from within the right visual field. This result approximated similar studies of animals found significantly more reactive to novel and potentially threatening stimuli viewed predominately with the left eye (right brain hemisphere specialization) than with the right eye and the opposite side of the brain [2,30].

A subsequent video recorded study of over 165 individually identified dairy cows also provided a choice for passing a human within the right or left visual field in what was termed a Forced Lateralization Test (FLT) [32]. Here the experimenter stood motionless and silent in the middle of the laneway, facing the approach of the cows as they returned to their home paddocks after afternoon milking at a university dairy. Cattle were repeatedly trialed in two series of experiments, firstly with the experimenter wearing the same coloured overalls as typical university students to present a comparatively familiar stimulus. In the first FLT series, of the 138 cows that could be positively identified in 11 daily trials, most demonstrated a significant overall right-eye preference to the stimulus (70%–90% passed the experimenter within their right-eye visual field each day except for an outlier result on trial 9 of 54% right-eye preference [32]). In the second series of FLT trials the experimenter wore overalls of an unfamiliar khaki colour, hat, sunglasses and a nuisance mask to cover the nose and mouth to increase the novelty value of the test stimulus. The cows showed a significant group-level left-eye preference to this novelty only on the first day of trials (24% passed the experimenter within their right visual field), and this preference dropped to non-significant ambipreference for most of the remaining 14 days of trials [32]. While a significant group-level left-eye preference was observed on two other days of the trials, significant right-eye preference for passing the unfamiliar masked experimenter did not occur [32]. Thus, at the level of the group, a significant preference to view an unfamiliar stimulus within the cow’s left visual field was found to be strongest for the first exposure to a completely novel stimulus, in findings corresponding with those from the herd-spitting experiments [15,32].

Most interestingly, researchers were able to identify two cohorts of cows that possessed either consistent left-eye preference (LE cohort) or right-eye preference (RE cohort) when responding to either an unmasked or a masked experimenter throughout both FLT series [32]. Cows in the LE cohort were found to have significantly higher scores than their RE cohort counterparts when assessed for restlessness in a standardized test of restraint [32], suggesting elevated levels of stress. The same large group of dairy cows were retested two years later in another FLT test involving a new unmasked experimenter as a test stimulus [33]. Here, an overall right-eye preference was found for cows passing the experimenter in a manner that remained consistent across nine days of trials that replicated the findings of the earlier study [32]. Cows from the earlier RE and LE cohorts were traced through the trials and found not only to have viewing preferences consistent with the previous study, but also possessed different patterns of characteristic behaviour when passing the unmasked experimenter [33]. Cows in the RE cohort were more likely to do so in pairs and to turn and look at the human when passing. By contrast, cows in the LE cohort were more likely to hesitate, sniff the ground, walk slowly, defecate whilst passing, pass singly and without turning their heads towards the person [33]. In restraint tests, cows in the LE cohort returned higher flight scores than cows in the RE cohort, supporting observations that the LE cohort demonstrated appreciably higher levels of stress across all behavioural assessments [32,33].

Cows in the LE cohort from the FLT experiments were found subsequently to have a significantly higher milk yield to their RE cohort counterparts [33]. This finding complemented those from an earlier study which was the first to indicate lateralized processing in domestic cattle—also the first to suggest a direct relationship between lateralized cognition and autonomic regulation in any vertebrate [34]. The lateralization was revealed in an analysis of production data from Russian dairies in which Holstein cows were confined indoors in individual stalls [34]. After being approached and fed a high quality ration from either the left or the right side daily for as little as 4 months per year, historical production records showed that cows approached and fed from left side produced nearly 10% more milk, had shorter service periods, produced more calves and were culled later than their matched counterparts fed from the right side (Table 1: [34]). Increases in these production measures were generally sustained over the remaining 8 months of the year, during which time they were randomly fed from left or right sides. Cows approached and fed from the left and not right side were found to remain significantly more productive in all measures, except milk volume, when fed a relatively poor quality ration diet compared with that of a normal dairy ration [34]. In a cohort that had their direction of feeding approaches reversed from left to right, milk production volumes were significantly reduced by comparison to cows that had feeding approaches changed from right to left [34]. One possible explanation for the significant discrepancies in left- over right-side fed cows [34], and left over right visual field monitoring [33], is that left-eyed cattle possessed moderately elevated sympathetic nervous system tonus and this supported elevations in milk production and fecundity—but this hypothesis remains to be tested. However, the authors attributed the lateralized production outcomes to asymmetries in motor postures in the cattle anticipating the good quality feed [34], as the behavioural response is guided by visual processing it is included in the summary Table 1.

To date, the range of responses discussed describes lateralized activities in cattle groups responding specifically to a human stimulus or husbandry management. In the first report of cows tested individually for lateralized responses, researchers studied the responses of 216 dairy cattle to three sets of novel objects placed bilaterally or centrally within a narrow race [35]. Responses were scored from the cows in single trials for three consecutive days for responses to a centrally positioned Kong (hollow blue toy for dogs) suspended above head height and matched experimental stimuli positioned bilaterally on the inside walls of the race. These latter novel objects consisted either of black and white checkerboards or small yellow balloons in successive series of 3-day trials [35]. The response behaviours that were scored included cows viewing preference/eye use, exploration behaviour as judged by which nostril used to touch an object, and whether the cows halted at distance before proceeding to closer inspection [35]. The number of cattle responding to the stimuli generally declined over the days of each test. No significant eye or nostril preference was found for investigating the centrally positioned Kong toy. However, a significant right-eye and right nostril preference was found overall for viewing and exploring (with their nose) the right rather than the left checkerboard or balloon [35] and this preference was strongest for the cows that had approached the stimuli directly without hesitation. By contrast, the cohort of cows observed stopping at distance exhibited a left side preference for subsequent close inspection of the yellow balloon (27 left, 11 right balloons touched with noses over 3 days of trials [35]). The pattern of response lateralization across the group suggested that while most cows rapidly familiarized with the test stimuli in a process that suggested right-side (left hemisphere) processing, a small proportion of cows exhibited heightened caution associated with predominantly left-eye (right hemisphere) processing.

Cows tested for lateralized visual responses to a motionless experimenter in the FLT tests were also assessed for evidence of lateralized social behaviours [32]. Success in agonistic interactions between any two cows within a herd of 233 were used to develop a dominance hierarchy across 992 scored observations. Cows scored as having lost on any encounter were found to have a significant left-eye preference for viewing the victor, and this was true of both the older cohort and the submissive losing cohort [32]. No overall population lateralization was found in this study of lactating cows, in contrast to strong left-eye lateralized responses for agonistic encounters in smaller horse bands of mixed sexes (feral horses and Przewalski horses in respective reports: [36,37]). Interestingly, however, two contrasting patterns of lateralized eye use emerged from the study that supported two different but consistent sets of behavioural strategies that the cows expressed across different contexts [32]. Cows that predominantly used their left eye during agonistic interactions were found to be more likely to use their left eye when approaching and closely passing a novel person wearing a nuisance mask in forced lateralization tests and also found to have significantly greater numbers of reactions or flight responses scored—suggesting that the left-eyed cattle were generally more aroused or anxious within test conditions [32].

In each study involving choice tests outlined above, there appeared, however, to exist a cohort of individual cows that appeared consistently and strongly left eye/right hemisphere dominant for viewing novel stimuli, while also characteristically exhibiting more cautious and flight-associated sets of behaviours, and, where measured, a significant tendency to lose agonistic social contests. One possible explanation for this dominance in responses directed by the left eye (right hemisphere) in such a cohort may be that this group of cows was predisposed to negative cognitive bias [38]. Recent reviews in the field of lateralized animal cognition have identified a range of broad cognitive styles that might be attributed to the respective brain hemispheres (e.g., [38]). Negative cognitive bias relates to the self-protective roles served by the left hemisphere and the dominance in sympathetic nervous system responses to potential stressors. By contrast, positive cognitive bias relates to the functions associated with retrieving positive memories or making positive judgments associated with the expectation of comparatively rewarding outcomes [38,39].

Overall, however, the studies of visual processing in cattle have demonstrated that while a generally lateralized process exists for left eye/right hemisphere dominance for initial processing of the novel properties of a given stimulus, there exists a demonstrably rapid process of learning and familiarization that reverses the preference to right eye/left hemisphere dominance. This directional right-to-left shift in hemispheric processing supports previous studies of animal learning and the consolidation process for long-term memories (summarized in [6]). Therefore, stimuli to which cows have fully habituated towards are probably paid minimal subsequent attention—given the declining numbers of scores demonstrated in various experimental studies (e.g., [35]). Rapid and efficient learning processes would appear to be a beneficial attribute for the domestication of large livestock, and with particular regard to the safety of the human handlers.

## 3. Cattle Domestication and Early Religious Symbolism

The Aurochs (*Bos primigenius*) was an early bovid that ranged across Europe and North Africa, the Middle East, and into Asia and formed the progenitor of the domesticated *Bos tauru*s and *B. indicus* breeds. Current evidence indicates that Aurochs most likely originated within the Indus Valley before occupying a maximum range extending East–West across the Asian and European contingents from coastal China to the British Isles, generally within a North–South approximately 3000-km-wide in a temperate zone and including both Northern and Southern (African) borders of the Mediterranean Sea (e.g., [40,41]).

The extinction of the Aurochs occurred in Poland in 1627, but the species held the distinction from contemporary writers as being untamable and, although reportedly benign to human activities, would aggressively target and kill humans when provoked (e.g., “...These are a little below the elephant in size, and of the appearance, colour, and shape of a bull. Their strength and speed are extraordinary; they spare neither man nor wild beast which they have espied.” Julius Caesar, *The Gallic War*, Book 6 Part 28, 53 BCE cited in [40]). 

The earliest identified example of *B. primigenius* comes from an isolated cattle mandible found in a Northeastern Chinese riverbed dated to 8660 BCE (10660 BP: [41]). Dental wear indicated habitual oral stereotypy, such as ‘cribbing’, as occurs in penned animals, rather than wear typical of use of a bit and halter [41]. While a complete mitogenome was able to be phylogenetically analysed from the mandible, the results indicated a novel Auroch lineage indigenous to China, an outlier example as no other cultural or genetic material has been found, suggesting a significant domestication event [41].

Recent sequencing and analysis of the complete mitochondrial genome of seven Indian cattle breeds indicates that in addition to the previously known domestication of a sub-species of Aurochs within the Indus Valley around 6500 BCE, another domestication location in Southern India also gave rise to the humped zebu cattle: *B. indicus* [42]. A earlier assumption that *B. taurus* arose from a point source domestication of Aurochs around 8500 BCE within the Fertile Crescent—the region of the Tigris and Euphrates Rivers within Iran and Iraq extending east across from the Persian Gulf towards and southwards along the eastern border of the Mediterranean Sea—has been disproven in recent studies [43,44,45,46,47,48]. Genetic analyses of modern breeds of European *B. taurus* cattle showed that multiple domestication and hybridization events likely occurred to reveal a complex pattern of interaction as the process of domestication spread east across the European continent. For example, a recent study of mitochondrial DNA from identified a second site of Auroch domestication within the Balkans north of the Taurus Mountains and Fertile Crescent [49].

Several sites across the Middle East show persistent use for community feasting on Aurochs and hunting over a long period of time during the pre-pottery Neolithic period (8600–6700 BCE) [50]. Together with a range of studies, a pattern of cooperative hunting, community feasting and ritualistic practices support the premise of an early “cult of the bull” by farmers early in the agricultural revolution taking place in the Middle East and Asia Minor [51]. The role of Auroch symbolism for funeral and mortuary functions in early Middle Eastern cultures appears particularly important [51]. The structure of Auroch use in the archaeological record changed gradually throughout time, presumably as the impact of domesticated cattle supplanted many roles [49], the Aurochs increasingly scarce but the symbolism of their horns and bucrania featuring heavily in many communal structures dated at that period [52].

Due to its association with the Aurochs, the domestication process of cattle has been defined as a “prey pathway”, similar to that of goats and sheep that were domesticated earlier (e.g., [23]). This is in contrast to the “commensal pathways” of domestication shared by dogs, pigs, cats, and chickens and the later “directed pathways” seen for horses, donkeys, dromedaries and Bactrian camels [23]. In those latter examples, specific breeding aims had been developed from the understanding gained from earlier domestication events [23]. However, the domestication of cattle is somewhat unique in that although cattle became regarded for their meat, hide, milk, and traction, they were originally bred for religious and sacrificial roles [50,53,54]. The oldest evidence for dairy and traction roles served by domestic cattle is the discovery of a ceramic bull with a churn on its back, found in the southern Levant and dating around 4000 BCE [23]. Direct evidence of when the practice of using cattle for traction is currently unavailable, but it possibly started with cattle pulling carts in ritual processions [53].

### 3.1. Cattle in Early Religion

Domestic cattle—more than any other non-human animal—have served key associations with deities in ancient religions of the Middle-East. The religious beliefs of the peoples of Canaan are of particular interest as they also played an important role in the etymology of the modern Western alphabet, as will be outlined in later sections. Canaanites represented a mixture of semi-nomadic and settled tribes from the region of today’s Lebannon, Israel, Palestine, Jordan, Syria, and parts of Iraq and Saudia Arabia [54]. Canaan was central to the Middle Eastern region historically referred to as the Levant. Canaan occupied a geopolitical centre between Egyptian, Hittite, and Assyrian (Mesopotamian) empires most significantly throughout the Bronze Age (3300–1200 BCE), however, was replaced by the development of Hebrew and Israelite identities during the subsequent Iron Age.

The Canaanite supreme god and father-figure to a range of lesser deities, and creator of man and all other animals, was called *Toru-’El*, or *’El* [54]. The apostrophe (’) denotes a glottal stop sound, used in Western and Northwestern Semitic languages—ancient Arabic (Northwest Semitic branch), Hebrew, Aramaic, and Canaanite-related cultures, including the Amorites from the city-state of Ugarit, and later the Phoenicians. *’El* was usually described with the attributes of a bull, being strong, and fertile and portrayed with a bull-horned headdress in human form, or simply depicted as a bull [54,55]. Earliest recordings of *’El* were found described in the archaeological site of the Tell Mardikh Royal Library in Syria dated to 2300 BCE. *’El* is a Northwest Semitic word meaning “god“ or “deity”, and is derived from the archaic Semitic ’l, a biliteral word for “god“.

**Figure 2:**
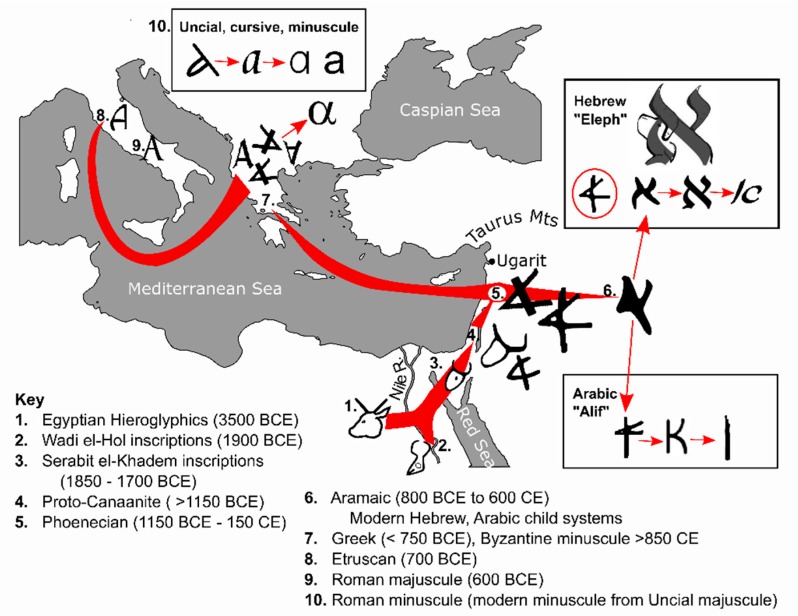
Ten key stages from archaeological and anthropological evidence leading to the modern letter “A”, or alpha. The location of the Taurus Mountains and the city state of Ugarit are also marked on the map as these are locations discussed at length in the text. The Canaanite/Phoenician city of Byblos is located beneath point “5” on the Mediterranean coast. (1) Egyptian hieroglyphics are thought to have served as a model for the Canaanite abjad or alphabet. (2 and 3) The stages of development of the Canaanite are defined as those predating 1150 BCE found outside of the ancient borders of Canaan as “proto-Sinaitic”, and (4) those within Canaan as “proto-Canaanite”. (5) Inscriptions found dated between 1150 BCE and 150 CE within Canaan are classified as “Phoenician” or “Canaanite”. (6) An early derivative of the Canaanite abjad was the Aramaic—not represented geographically—which was later used to form the basis of modern Hebrew (top inset) after replacing an earlier palaeo-Hebrew script based on Phoenician (shown encircled). The underlying ox-head viewed from the left side is illustrated for the majuscule Eleph and the modern minuscule Eleph is also presented. The Aramaic abjad also formed the basis for the Arabic abjad, as shown in the lower inset, progressively reduced over time to the single stroke for the modern ’Alif. (7) Ancient Greeks adopted the 22 letters of the Phoenician abjad and added or replaced four symbols for vowel sounds, recycling the glottal stop sign of ’aleph for its current Western use, alpha. A practice later developed in different regions of Ancient Greece to orient alpha in ways that designated the regional origin of the writer. This was illustrated stylistically, not geographically in the Figure. An uprighted (Ionian) orientation for alpha was formally adopted across Ancient Greece. A minuscule alpha was developed later with the introduction of Byzantine script (arrowed offshoot). (8) Trade between Greece and the Etruscan civilisation led to the adoption of the alphabet on the Italian peninsula, however, the Etruscans were the first to standardize the left-right direction for writing. (9) The Roman tribe adopted the Etruscan alphabet and writing practices during successive conquests of Italian tribes to form the Roman Republic across the unified Italian peninsular. (10) Roman (Latin) minuscule developed gradually from informal and cursive handwriting style (circa. 200 BCE–600 CE, examples not shown). An “Uncial” style of lettering was developed by Roman clergy and scholars and was in use around 100–200 CE, the alpha symbol clearly recapitulating ancient Phoenician forms for ’alep. Together, Uncial and early Roman cursive form the basis for the modern minuscule (lower case) script.

Of *’El*’s children, *Baa’al Hadad*, or *Hadad*, or *Ba’al* (meaning “Lord” or “owner”) is considered the most important—particularly for the Phoenician culture [55]. *Ba’al* is also portrayed with bull-like characteristics, including a horned headdress when not illustrated as a bull, and was also considered a “storm-god”, similar to the roles served by older gods from the neighbouring culture of Mesopotamia. *Ba’al* and his wife *’Anat*, the Canaanite warrior goddess, had their home on Mount Zaphod (now Mount Sapan, or Jebel Aqra), in what is now called the Nur mountain range, branching southwesterly from the main Taurus Mountains complex and approximately 30 km north of the Amorite (Canaanite) city state of Ugarit (see Figure 2).

*’El*’s consort and sister *Ba’alat* (“Lady” or “Mistress”, also known as *Ba’alat Gebal* or *Baalat*) was typically depicted as a relatively more benevolent cow-like deity and became known as “The Lady of Byblos” [56], Byblos being a key Canaanite (Phoenician) city south of Ugarit. *Ba’alat* was considered the patron goddess of Byblos’ main trade products of papyrus, ceder, and semiprecious gems, such as turquoise, which were particularly highly valued by the Ancient Egyptians. *Ba’alat* also shared key characteristics with *Hathor*, Egypt’s creation goddess, such that they became considered as the same individual in a process known as syncretism [56]. Generous donations to the Temple of *Ba’alat Gebel* from the Pharaohs, particularly during the Old Kingdom period (2686–2130 BCE), helped to cement relationships between the two cultures with their shared cow goddess [57].

Cults of sacred cows and cow goddesses were prevalent throughout the Middle East during prehistoric times. However, before the advent of Egyptian hieroglyphics in around 3000 BCE, it is not currently possible to attribute unnamed carvings of beautiful goddesses with cow horns or cow ears before the Ancient Egyptian First Dynasty (5000–3150 BCE) to *Hathor* or a similar goddess *Bat*, who *Hathor* replaced by syncretism [56]. *Hathor* (like *Bat*) was a sky-goddesses with various mythologies, including the Milky Way galaxy representing the goddess’ body, or spilled milk from her breasts [58]. The stars of the Milky Way galaxy were considered to represent the souls of *Hathor*’s children that were awaiting birth or to be with her again in the afterlife. In human or cow form, *Hathor* is depicted wearing a crown of cow horns, cradling the disc of the sun or the moon. *Hathor*’s name literally means “mansion of *Horus*” or “house of *Horus*”, and is thought to refer to the entire sky across which travelled her consort (or son) *Horus*—a Sky and Sun God [56,58]. She is often referred to as "The Golden One" because of her association with the sun, and in later incarnation cycles became both the mother and the daughter of the Egyptian Sun God. *Ra*. *Hesat* is another cow goddess, typically portrayed as a white cow representing purity, her milk giving life to humanity [56]. *Hesat* is widely viewed as one of *Hathor’s* manifestations. *Hathor* became a key Egyptian deity over a 6000-year period as the supreme goddess of music and dancing, joy, love, sexuality, fertility, and the afterlife [56,58].

## 4. Text and Context—Starting the Alphabetic Code

The discussion below only relates to the key stages leading to the use of the ox-head to represent the first letter of the modern alphabet: the letter A. The earlier cuneiform script of Sumer, Mesopotamia, and the scripts of the Ugarit city state and Crete were ignored, as were the Chinese and Japanese syllabaries and logograms, as they developed independently of the modern Western alphabet.

It is worthwhile to first briefly outline the etymology of Western phonetic coding systems. The first lexicographical systems included only consonants and relied on the reader inferring the appropriate vowel sound. Such a consonantal ‘alphabet’ is termed an ʾabjad (أبجد, spelled from right to left), named after the initial ’alif (أ), beta, gamma and delta letters of the Arabic and Hebrew script [59,60]. Note that the first letter was not the modern vowel sound for alpha, but the glottal stop sound as used in Semitic languages. ’Āleph is the first letter of the Semitic abjads, including Aramaic Ālap, Syriac *ʾ*Ālap̄, Phoenician *'*Ālep, Hebrew *'*Ālef or Eleph (א), and Arabic ’Alif (أ). Some subsequent consonantal phonetic scripts became elaborations of the abjad, and these are termed abugida—an Ethiopic word applied to writing systems whose basic characters denote a consonant followed by a basic vowel, with other vowels being marked by modifications or appendages to the respective consonant [59]. Modern Ethiopian (Ge’ez) script and also most of the scripts of India are examples of abugidas, such as the Devanagari script used in India and Nepal. The first true “alphabet” was developed by the Ancient Greeks, using specific symbols to represent the five vowel sounds as found in the 26 letters in the modern Western alphabet [59].

The following ten stages are summarized graphically in Figure 2. The approximate dates provided for each stage reflect the current consensus from archaeological research [60]. A number of historical conventions were developed to distinguish comparatively few examples of, but nonetheless similar lettering systems found throughout the Middle East inscribed on rocks, bronze arrow and javelin heads, clay tablets or pottery. Figure 2 provides a small range of exemplar ox-head symbols from Proto-Sinaitic, Canaanite and Phoenician inscriptions that reflects the range of variability that has been found. These variations in styles were conventionally classified by geography and arbitrary time periods which, in some cases, could not be dated accurately. All the examples were found in scripts written predominantly in a right-to-left direction. It is noteworthy that only four animals are represented symbolically within early developments of what became the 26 letters of the modern alphabet—the ox, fish (F), snake (N) and monkey (R) [61].

### 4.1. Egyptian Hieroglyphics (3500 BCE)

The hieroglyphics of Ancient Egypt are comprised of a complex mixture of logograms—symbols representing concepts or ideas (also commonly termed ideograms or pictograms), syllables, and phonemes. Depending on the context, a hieroglyph could be used as either a logogram, a syllable or a phoneme [62]. Ancient Egyptian phonemes are symbols representing 24 consonants, such as used in the modern Western alphabets—however, no vowel sounds were represented [59,63]. In addition to the formal paintings and carvings, hieroglyphs were used for important texts, such as The Book of the Dead. Animals, deities and human figures used in Egyptian hieroglyphic sentences were conventionally depicted facing towards the start of the sentence, however there was no established convention in the direction of writing [62]. Therefore, symbols faced left for left-to-right sentences, or right for right-to-left sentences, or faced a uniform left or right direction when written in the vertical. The symbol of a bull’s head viewed from the side was used by the Ancient Egyptians as a logogram to represent the concept of ‘wisdom’, and not as a phoneme [62].

Hieratic script based on the hieroglyphic symbols was developed around 3000 BCE as a more convenient form of writing involving a simplified cursive script. Hieratic script was chiefly used for everyday administrative written communication on papyrus using ink and reed pens. A lapidary format also existed for leaving messages and graffiti on rock surfaces. By convention, a horizontal right-to-left direction of writing was predominantly used for Hieratic script until about 660 BCE. At that time, Hieratic was overtaken in popularity by Demotic script, which was based on Hieratic script and written right-to-left [62].

### 4.2. Semitic Inscriptions at Wadi el-Hol (1900–1850 BCE)

**Figure 3:**
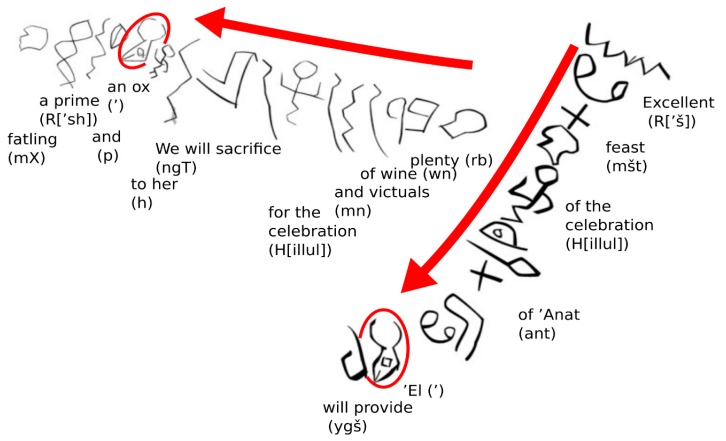
Wadi el-Hol inscriptions. This interpretation implies a message between allied forces, possibly a mixture of Egyptian and Canaanite soldiers [64,65,66]. The inscription includes symbols strongly reminiscent of letters from the early Phoenician (Canaanite) abjad or alphabet, and Egyptian hieroglyph symbols for celebration. Footnote: Interpretation of two sections start with the vertical section top-to-bottom: “Excellent (R[’š]) feast (mšt) of the celebration (H[illul]) of ’*Anat* (ant). ***’El* (’)** will provide (ygš)...”, and completes with the horizontal section right-to-left “...plenty (rb) of wine (wn) and victuals (mn) for the celebration (H[illul]). We will sacrifice (ngT) to her (h) an **ox (’)** and (p) a prime (R[’sh]) fatling (mX)” [66]. Note the leftwards facing ox-heads encircled in the figure and emphasised in the previous text. The first ox-head is used as a phoneme letter ***’****El*, and the second is used as a logogram (“calf”). The latter faces in the direction opposite to the convention for Ancient Egyptian hieroglyphs to face toward the start of the sentence. The text describes words for Canaanite/Western Semite gods—*’El* the bull-like supreme creator and *’Anat* the warrior-goddess.

Currently, the earliest-dated example of script using discrete symbols as phonemes is that of the Wadi el-Hol inscriptions, carved into a cave wall alongside known routes of Ancient Egyptian armies and their Canaanite allies. Originally discovered in 1993 between the ruins of Luxor and Thebes in the Western Upper Nile, a subsequent study dated their age to 1900–1850 BCE, based on the nearby hieroglyphic and lapidary hieratic inscriptions mentioning contemporary Pharaohs and other notables [64,65]. The message appears to be an early example of graffito and reads in two sections in top-to-bottom vertical and right-to-left horizontal sections, to use the available rock face effectively [64]. Figure 3 shows an annotated reproduction of the complete message.

The Wadi el-Hol inscriptions show great similarity with a number of symbols found in 22 consonants listed in the first abjad known to be commonly used—that of the Phoenicians, descendants of the Canaanites. The text differs from the later abjad in that figures are used to indicate jubilation or celebration—just as they are used in Egyptian hieroglyphic and hieratic scripts [64]. One interpretation of the inscription [66] indicates involvement of Canaanite scribe and audience, as the text uses words for Canaanite/Western Semite gods—*’El* the bull-like creator and *’Anat* the goddess of war.

The use of the ox-head symbol is applied in two different contexts—not only to represent a glottal stop sound, but also pictorially to represent a calf—similarly to the manner in which Egyptian hieroglyphs were used [66]. Although the Wadi el-Hol inscriptions represent a single example of the use of an early alphabet or abjad, there is a significant break from Egyptian tradition in that the ox is not shown facing the start of the sentence [64]. Rather, the left side of the ox head is displayed in the right-to-left sentence (see Figure 3). The symbols are in lines and not grouped in quadrants, as is common in lapidary hieratic of the period [64]. The Egyptian meaning of “wisdom” for the ox head logogram is also not applicable here. Thus, inscription represents an important transitional phase before the complete removal of logograms, such as the “calf” and “celebration” symbols, from what was to become an entirely phonetic lettering system of writing.

### 4.3. Inscriptions at Serabit el-Khadem (1850–1700 BCE)

Over 30 examples of inscriptions on rocks and statues using early Canaanite abjad symbols have been found in the exhausted Egyptian turquoise mine of Serabit el-Khadem, in the southern-central Sinai peninsula. These has led to the classification of a “proto-Sinaitic” script form, which is conventionally used to describe all examples of pre-1150 BCE Canaanite script found outside of Canaan. Thus, the Egyptian inscriptions at Wadi el-Hol (item 2, above) is also considered Proto-Sinaitic [65,67].

The Serabit el-Khadem mine is located near an earlier temple to *Hathor*—the Egyptian supreme goddess of celebration and the afterlife. *Hathor* is also the patron deity of miners, and many inscripted artefacts from the temple site suggest offerings from Canaanite miners. In one example of syncretism, a small Eygptian sphinx-like statue dated to around 1500 BCE found at *Hathor*’s temple site is inscribed with the Canaanite “To the Lady” (*Baa*’*al*—the cow-goddess of Byblos). The sphinx is also inscribed with Egyptian hieroglyphs that read “beloved of *Hathor*, mistress of turquoise” [63,66]. Several other Egyptian lapidary inscriptions from the site describe the goddess *Hathor* as “the lady of turquoise” [63,66].

### 4.4. Proto-Canaanite Script ( >1150 BCE)

Figure 2 provides two different examples of the ox-head symbol used in inscriptions dated to before the completion of the Bronze Age Collapse (1200–1150 BCE). Due to variability in the comparatively few examples of inscriptions found with reliable dating within Canaan, there is no commonly supported transition of styles that distinguishes proto-Canaanite from later Canaanite and Phoenician scripts [67,68].

### 4.5. Phoenician Abjad (1150 BCE–150 CE)

The earliest complete abjad is Phoenician, containing 22 symbols. Ironically, this representation is written in Ugaritic cuneiform, as no complete example has been found inscripted or written on papyrus—the preferred writing medium used by the Phoenicians. Papyrus failed to survive in the comparatively humid conditions of the Mediterranean, in contrast to papyrus examples found in Egyptian tombs (e.g., [69]). Our current knowledge of the Phoenician religious beliefs and lettering comes from the northern Amorite Canaanites in the northern city state of Ugarit, through Ugaritic cuneiform written in clay tablets (e.g., [69,70]). Ugarit possessed an abjad of 29–30 cuneiform symbols based on the Phoenician abjad (e.g., [69,71]), which places ’alep at the start of the lexicographical list. Interestingly, Ugaritic was conventionally written from left-to-right, a Babylonian tradition [69]—in contrast to other Semitic cultures preferring to write in the opposite direction, or without a conventional direction for writing in the horizontal. Some later examples of text are oriented right-to-left, indicating more Phoenician influences over time (e.g., [69]). Ugarit was destroyed during the Late Bronze Age Collapse and Ugaritic cuneiform fell into disuse [72].

### 4.6. Aramaic (800 BCE to 600 CE), and Child Scripts of Hebrew and Arabic

The Amaraic Canaanite language initially used the Phoenician script but gradually changed in appearance over time. Variations of the Aramaic abjad were subsequently adopted by other Semitic cultures, including Hebrew and Arabic. Ancient or Paleo-Hebrew was originally based on Phoenician. However, by 500 BCE, an Imperial Aramaic was used by decree of the conquering Persian Emperor Darius I [60]. A “square” style script based on Imperial Aramaic was later adopted for modern Hebrew and a cursive variant was also developed [72].

In 600–500 BCE, Aramaic abjad was also adopted by the Nabataeans—a northern Arab tribe with a Northwest Semitic language. Different styles of lettering were subsequently used for inscription and for writing on papyrus, and by 100 CE, a unified Nabataean abjad with characteristics from both written forms was adopted as the Arabic abjad [72]. Branches of the Aramaic scripts were later adopted by early subcontinental Indian scripts [73].

### 4.7. Greek Alphabet (>750 y BCE)

The Phoenician abjad was subsequently adopted by the Ancient Greeks, who introduced some new innovations. They had no use for glottal stops in their language, and instead, exchanged some abjad symbols for vowels and added some other symbols of their own to complete the first true alphabet [74]. This included the use of the “ah” or “ay” vowel sound to be represented by the ox head-shaped first letter—which they called “alpha” instead of “aleph” [73]. While the left-sided ox head was adopted by the Greeks to depict the first letter, regional differences in a range of letters became conventionalised to indicate the origin of the writer. Thus, different Greek states rotated the ox-head clockwise (uprighted), inverted anticlockwise and even reversed to face the right [75]. The uprighted alpha and other letters of the Ancient Greek alphabet gave rise to Coptic, Gothic, Armenian, Cyrilic and Georgian scripts (not shown in the Figure).

Writing direction in the horizontal was not standardized for Ancient Greek and could be left-to-right, right-to-left, and popularly for inscribing on objects that could be turned in the hand, in *boustrophedon* (“as the ox turns”. Boustrophedon is a type of bi-directional text where every other line is flipped and/or reversed so that alternate lines are read in opposite directions [74]. The modern left-to-right writing direction was standardized in Greece around 500 BCE [74]. Minuscule letters were adopted from the Byzantine script in around 835 CE [74], where the Greek minuscule alpha (α) is currently used conventionally for scientific and mathematical notation.

### 4.8. Etruscan Alphabet (700 BCE)

The Etruscans were an Asia Minor peoples that had settled in a region of Tuscany on the Italian peninsula. Through their trading networks, the Etruscans adopted their lettering from Greek traders, including the up-righted alpha [61]. While some Etruscan texts were written in boustrophedon style, like the Greeks of the time, Etruscans were significant for being the second culture to conventionally use horizontal writing from left-to-right, after the Ugarit cuneiform.

### 4.9. Roman/Latin Script (600 BCE)

Roman conquest of the Etruscans and other Italian cultures led to the unification of the Italian peninsula and the formation of the Roman Republic. The Etruscan writing system was adopted and used throughout the expansion of the Roman Empire [61].

### 4.10. Roman/Latin Minuscule Development

Subsequent modifications to the modern alphabet were Roman innovations. While popular and used extensively in permanent stone monuments—such as the ‘Trajan’ font—Roman majuscule was slow to write and impractical for recording speeches and political debate. Informal, cursive handwriting scripts called “Old Roman Cursive” (circa. 200 BCE–200 CE) and “New Roman Cursive” (200–600 CE) for everyday use of reed or quill on paper were developed, the latter forming the basis for the modern minuscule (lower case) script. Examples of these cursive styles are not shown, however a cursive “Uncial” style was developed about 100–200 CE, and together with New Roman Cursive, formed the basis for the modern compact cursive and minuscule (lower case) alphabet [61]. The left-side ox-head was again reutilized for the majuscule Uncial alpha and clearly defined in the minuscule alpha.

## 5. Discussion

There is no doubt that the domestication of cattle was a significant achievement in early Western civilisation. The role of cattle seems first to have been revered primarily as religious instruments, and consequently celebrated in the primary position within the Western phonetic alphabets for over 4 000 years. This position has remained stable while the value of cattle shifted from sacrificial roles into the commodities of milk, meat, hides, and tractor power. The word ‘cattle’ is an Anglicised version of “chattel”, regarding the comparatively later consideration of domestic cows as currency. The following discussion outlines the key points of the hypothesis that early recognition of cognitive lateralization in domesticated cattle played a significant role in the symbolism that has been carried forward in the modern letter ‘A’.

### 5.1. Conservation of the ‘Alpha’ Position

There has been previous debate in the literature about the strongly conserved position of the ox head to lead the alphabet across the breadth of Western languages, and certainly simple traditional conservatism alone could explain its maintained location at the forefront of lexicography [70]. Additionally, the interchangeability of letters with numerals may have helped to preserve such conservatism such that the ox-head also represented the number ‘one’ at various periods of Ancient history. Examples include the use of the alphabet as numerals by the Ancient Greeks from about 700–400 BCE [76,77], and this custom was followed by the utilization of the respective Semitic abjads of the Ancient Hebrews for a quasi-decimal numeral system and the Ancient Arabians for a decimal numeral system. In the latter instance, the Arabic abjad numerals were used in the Arabic-speaking world before approximately 800 BCE when Hindu-Arabic numerals were adopted [77]. While the first-place position in the Western alphabet was maintained by the ox-head despite considerable shifts in successive Semitic and European cultures that employed its use, an explanation as to how it was awarded that position may rely on the role of cattle in early religions. Section 3.1 above briefly addressed the bull-like Canaanite creator *’El*, a name that became synonymous with "god". It would appear likely that should the ox-head ’al represent not only a glottal stop, but also a shorthand symbol for god, then such a symbol might therefore be expected to be placed first before all else in any early religious society.

### 5.2. Early Religious Significance

The “cult of the bull” hypothesis raised by Cauvin [78] presents early religions as significant socio-political forces in the neolithic Middle East, and probably interconnected with ongoing domestication events of both plants and animals. Positive confirmation is still lacking on the measurable impact of the birth of such divinities or cults had in the emergence of cultures the pre-pottery Neolithic (e.g., [79]). Nonetheless, in the initial stages of the transition from nomadic hunter-gathering to organized sedentary communities with selected crops, early religions likely helped to connect and unify an ever-growing populace with shared language and ideals. Early experiments to control wildlife to ensure reliable meat supply or demonstrate man’s growing mastery over nature are not precisely known, but the size, power and imposing horns of the Auroch undoubtedly made them a target for religious symbolism. The natural reproductive behaviour of the bull Auroch probably imbibed the powers of strength and virility to the male members of sedentary communities as their role transitioned from that primarily of hunters to that of herdsmen, farmers and tradesmen [80,81]. Symbols of leadership in the Middle East included not only Auroch horns and bucrania, but also phallic pillars and posts inscribed with symbols of oxen for community gathering places (e.g., [52]). Wild Aurochs still ranged in increasingly contracted regions around Middle-Eastern settlements and communal hunting and feasting on these animals particularly for funereal celebrations probably underscored their early religious symbolism (e.g., [50]).

In an early drive to monotheism, the Ancient Hebrew supreme god *Yaweh* was likely developed from the earlier Canaanite *’El* [55,82]. The Hebrews adopted “Isra-’*El*”—“god rules”—as the name of their nation, Israel [54]. However, left-sided ox-head survived as ’alp and ’eleph in Ancient Hebrew to represent concepts such as god, ox, strength and leadership [83]. As a surrogate shorthand for ‘god’, the meaning behind the left-sided asymmetry of the majuscule alpha was possibly lost to the broader populations of Ancient Greeks, Etruscans and Romans until scholarly Roman Catholic monks reintroduced the asymmetrical “A” as Uncial and later cursive and minuscule forms (see Figure 2). Perhaps for this reason, the majuscule and minuscule forms (A, a) are the most disparate of all the letters of the modern alphabet.

### 5.3. The Lateralization of Domestication

The early deification of wild and domesticated oxen probably occurred in parallel with a lengthy period of selective breeding of sacrificial animals with increasing docility and tolerance of close human handling. While selection for “docility” may have been the conscious selective aim of early breeding efforts, it is arguable that there was unconscious selection [26] for cognitive lateralization in the domestication process, as discussed in detail below. Indeed, docility may possibly be a side-effect of the possession of a suite of lateralized processes—a learned pattern of considered responses or coping strategies to mitigate stressful interactions with humans.

In Section 2 and Table 1 presented earlier, a range of cognitive and physiological functions found to be lateralized at the population level in domestic cattle was outlined. There is currently no obvious relationship between the domestication of various species and their expression of behavioural laterality, however. Species considered domesticated through a commensal pathway include dogs, cats, pigs, pigeons and chickens [23]. The domestic chick has served as model for non-human lateralization for decades [18]. By contrast, only comparatively recently have reports behaviour suggestive of lateralized cognition been reported for other commensally-domesticated species. These include dogs (summarized in [84]), cats (significantly more left-pawed than right-pawed cats, lateralized cats faster and more accurate than ambipreferent cats: [85]; male cats showed a significant preference for using their left paw while females were more inclined to exhibit a right-sided bias for spontaneous and targeted reaching behaviours: [86,87]) and pigs—which can also be considered within the group of prey domesticates (lateralization for agonistic contests was found absent at a population-level, but strongly lateralized pigs won their contests significantly faster suggesting that these animals were more efficient in their information processing: [88]).

A similarly complex pattern of lateralized behaviours has been reported for species considered as prey domesticates [23]. Only comparatively recently have reports of lateralization been published for reindeer (herd circling behaviour: [89], sheep (right-side bias to avoid an obstacle to join a social companion: [90,91,92]: day-old lambs were not found to have laterality for a range of simple behaviours: [93]), goats (right-side bias to approach and view images of smiling humans: [94]), and cattle (Section 2.2, above). For species considered domesticated by a comparatively directed pathway [23], only horses have yet been reported to possess a range of types of lateralized visual processes. Lateralized visual processing of stimuli that represent potential threats have been confirmed in horses in a range of conditions. When tested individually and approached from either monocular field by an experimenter with an umbrella opened towards the horse, a significantly greater reactivity in response was found for approaches on the left and not right monocular fields [95]. When approached from the frontal, binocular field, horses that escaped to the left side did so running further from the experimenter than those that escaped to the right [95]. Strongly lateralized preferences also exist for using the left eye/right hemisphere visual system for directing vigilance behaviours and initiating aggressive social interactions (feral horses [36]; Przewalski horses [37]). When interacting with humans, horses have been found to possess a left eye preference for viewing humans [31], and also a left eye preference for discriminating between positive (smiling) and negative (frowning) human facial expressions [96]. In non-production livestock, some selective traits (e.g., racing ability in certain horse breeds) may be acceptably traded off with forms of behaviour that may not otherwise conform with efficient lateralized cognitive processing, including unpredictability of temperament when handled.

The traditional use of the terms “near” for the left, and “far” or “off” for the right side of domestic livestock for over 300 years suggests a long, although informal, understanding of lateralized cognitive processing in cattle and horses [15,31]. Although the origin and explanation for these traditional terms is not currently known, it is likely that they represent a practical or functional relationship similar to the terms of “port” and “starboard” used traditionally to refer to the respective left and right sides of ships and other watercraft. Furthermore, in contrast to earlier anthropocentric theories as to the origins of “near” and “far” relating to human handedness [97], there is growing awareness that such terms rather reflect a “mutual convenience” for close contact between man, horse and cattle [15,31]. Given that interactions between humans and livestock (horses and cattle) can result in handler fatalities at an appreciably higher rate of incidence than other comparatively smaller domesticates, it is postulated here that in unconsciously selecting for lateralization, the domestication of cattle generated a species that was able to be predictable in its nature and comparatively safe when interacting with humans. More particularly, that unconscious selection for cognitive lateralization led to a domesticated species that could efficiently develop rules-based schema to better adapt to its comparatively artificial farming environment. Examples of efficient rules-based learning include simple cause-and-effect relationships, such as associating close human proximity with the provision of feed when corralled, permitting touching or grooming particularly when associated with receiving a food resource, and habituating to the use of a harness to be led. These are all types of experiences applicable to taming or gentling in a captive wild individual, however, in herd settings they could be related with the ability to learn socially or vicariously the experiences of a herd-member [98]. Moreover, these sets of adaptations appear to correspond with the positive cognitive bias that is attributed to left, and not right hemisphere functioning [38] (see Figure 1). This lateralized characteristic may have been particularly relevant within ritualized settings, as hypothesized early in the domestication process of Aurochs [53], where excluding actual sacrifice, presumably routine and therefore, anticipated rewarding events may have occurred. With regard to the social facilitation hypothesis of lateralization [2,20,21], any pre-existing forms of cognitive lateralization in the socially-organized Aurochs were probably co-opted or enhanced by unconscious selection to improve coping within the domestic environment.

Conclusions drawn from the open field challenge tests of cattle behaviour responding to an approaching or static challenge [15,29,33] included the assumption that individual cattle choose to view the environmental stressor with the eye system that provides the most effective mitigation or mediation of their internal physiological state. This is in keeping with theories of coping strategies [99,100], and implies that the animal is aware of its internal state and is also sensitive to the differences in analytical procedures specialized by either (visual) system. We could speculate that cows with comparatively higher levels of sympathetic tonus—prone to be more anxious than their herd-mates—are in readiness for flight and subsequently, more likely to maintain a vigilant left-eye watch of the human in the middle of the lane when passing (FLT: [32,33]). By comparison, cows that identify the same stimulus as non-threatening are likely to habituate more rapidly and use their left eye to monitor for other potential threats in the immediate environment. This possibility is supported by rapid switching of viewing preferences noted in adult chickens [101] and chicks [102] when challenged with an overhead predator model sighted initially with the right and not left eye. The switching of viewing preference for such stimuli does not occur in the opposite direction and indicates that the left eye (right hemisphere) provides the optimal processing system for predator stimuli because it is functionally coupled with the right hemisphere’s dominance of the sympathetic nervous system and flight/fight responses. In contrast, the Russian dairy study induced conditions in which significant environmental stimuli were routinely presented asymmetrically [34], and where there was limited possibility for half the herd to present the cognitive system best suited to the analysis. In such conditions, even relatively benign stimuli may act as significant stressors, as suggested by the significant differences in reproductive and productive output found between left-side and right-side fed cattle [34].

Cattle supplemented on a regular basis rapidly learn daily feeding times based on a possible range of visual cues (pers. obs.) and can even associate food rewards with spatial memories and entrained visual cues to enable self-herding without fences [103]. Recent advances with robotic milking demonstrated that cattle were able to learn appropriate strategies to mitigate their internal state—having their udders evacuated as required, and independent of larger herd movements, as occurs in traditional milking [98]. Cattle appear to prefer routine, and may actively seek temporal and spatial patterns within their environment in a drive for predictability and control [98]. The involvement of lateralized cognitive processing is not currently known for many such higher functions in cattle and other domesticates. Future studies in lateralized behavioural responses in domestic livestock will not only reveal detailed information about an animal’s cognitive processing, but also its affective state [39]. Mirroring human studies, animal models have been used in laboratory settings to show direct relationships with cerebral lateralization, the sympathetic nervous system, the hypothalamic-pituitar*y*-axis response system, and immunomodulatory control [104,105,106,107,108]. Further investigation of lateralized responses to chronic stressors have considerable promise for understanding and improving welfare conditions in cattle (e.g., [38]), particularly with regard to intensive management systems and reduction in antibiotic use, for example.

## 6. Conclusions

Knowledge of lateralized processing in domestic livestock is indicated not only from the traditional terms of “near side” for close handling livestock on their left side, and “far” or “off side” for the opposite, right side - but also indicated in the left-sided directional asymmetry of the alpha symbol. While this is evident today in the minuscule letter ‘a’, the antecedent majuscule letter ‘A’ was also depicted the head of a horned ox originally viewed from the left side. These examples collectively suggest a long history of lateralized cattle-human interactions.

## Figures and Tables

**Table 1:** Lateralized visual processing in domestic cattle. Summary of results showing significant cognitive lateralization or physiological responses to stimuli viewed predominantly with either the left or right eye. Interhemispheric transfer of information processing is also indicated by arrows, as illustrated in Figure 1. Not all cattle display the same pattern of lateralization when tested, and such cohorts highlight other key aspects of cattle behaviour. The traditional terms “near side” for the left, and “off side” for the right of domestic cattle and horses are included, despite lacking clear explanation for their use in over 300 years in the literature [29,31].

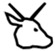	**Right Eye/Left hemisphere** “Off Side”	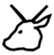	**Left Eye/Right hemisphere** “Near Side”
Rapid familiarization (habituation/learning) [15,32]	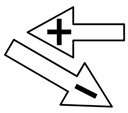	Novelty detection [15,32]	
Cohort * of cattle initiate flight in response to novelty [29] Variable responses [29]		Suppresses right hemisphere reactions including flight Permits close approach by humans Predictable responses [29,31]	
Most cows transition from left to right eye preference when passing a novel human repeatedly over multiple trials, indicating familiarization [32,33]		Cohort * persistently choosing to pass a novel human within their left visual field are comparatively more anxious on a range of other measures, although produce significantly higher milk yield than their counterparts choosing the right visual field [32,33]	
		Increased production associated with left-side feeding of high-quality ration nearly 10% greater milk volume shorter service periods increased calving rate longer productive lives [34]	
Most cattle tested without interrupting their approach from distance subsequently investigate static, novel stimuli (checkerboard, balloon) on the right and not left side when presented simultaneously on both left and right visual field [35]		Cohort * of cattle hesitant in approach subsequently choose to investigate static, novel yellow balloon on the left and not right side when presented simultaneously [35]	
More likely to dominate agonistic social encounters [32]		More likely to submit in agonistic social encounters [32]	

Contrasting behavioural responses are presented in rows. The first two rows of the table present summary findings from studies that correspond directly with the general pattern of vertebrate lateralization and effects of interhemispheric transfer [6], shown here as positive and negative arrows (see Figure 1). * Cohorts—small numbers of individual cattle and less than 10% of the respective study sizes [29,32,33,35]—have been observed with contrasting lateralized behavioural strategies, apparently failing to adequately familiarize to novel experimental stimuli. An alternative or complementary explanation may be that these cohorts of cattle express negative cognitive bias—a right-hemisphere dominant mode of processing discussed in the text.

## References

[1] Rogers L.J., Slater P.J.B., Rosenblatt J.S., Snowdon C.T., Roper T.J. (2002). Lateralization in vertebrates: Its early evolution, general pattern, and development. Advances in the Study of Behavior.

[2] Vallortigara G., Rogers L.J. (2005). Survival with an asymmetrical brain: Advantages and disadvantages of cerebral lateralization. Behav. Brain Sci..

[3] Ghirlanda S., Vallortigara G. (2004). The evolution of brain lateralization: A game-theoretical analysis of population structure. Proc. R. Soc. B Biol. Sci..

[4] Frasnelli E., Vallortigara G., Rogers L.J. (2012). Left–right asymmetries of behaviour and nervous system in invertebrates. Neurosci. Biobehav. Rev..

[5] Güntürkün O., Ocklenburg S. (2017). Ontogenesis of Lateralization. Neuron.

[6] Rogers L.J., Vallortigara G., Andrew R.J. (2013). Divided Brains: The Biology and Behaviour of Brain Asymmetries.

[7] Karenina K., Giljov A., Ingram J., Rowntree V.J., Malashichev Y. (2017). Lateralization of mother–infant interactions in a diverse range of mammal species. Nat. Ecol. Evol..

[8] Robins A., Phillips C.J. Visual preferences in cow-calf dyads: Lateralised vigilance and maternal bonding in domestic cattle. Proceedings of the 37th Annual Conference of the Australasian Society for the Study of Animal Behaviour (ASSAB).

[9] Freund N., Valencia-Alfonso C.E., Kirsch J., Brodmann K., Manns M., Güntürkün O. (2016). Asymmetric top-down modulation of ascending visual pathways in pigeons. Neuropsychologia.

[10] Duboué E.R., Hong E., Eldred K.C., Halpern M.E. (2017). Left Habenular Activity Attenuates Fear Responses in Larval Zebrafish. Curr. Biol..

[11] Maggs D.J., Miller P.E., Ofri R. (2008). Slatter’s Fundamentals of Veterinary Ophthalmology.

[12] Gibbs M.E., Andrew R.J., Ng K.T. (2003). Hemispheric lateralization of memory stages for discriminated avoidance learning in the chick. Behav. Brain Res..

[13] Robins A., Chen P., Beazley L.D., Dunlop S.A. (2005). Lateralized predatory responses in the ornate dragon lizard (*Ctenophorus ornatus*). Neuroreport.

[14] Robins A., Rogers L.J. (2006). Complementary and lateralized forms of processing in Bufo marinus for novel and familiar prey. Neurobiol. Learn. Mem..

[15] Robins A., Phillips C. (2010). Lateralised visual processing in domestic cattle herds responding to novel and familiar stimuli. Later. Asymmetries Body Brain Cogn..

[16] McGinley J.J., Friedman B.H. (2015). Autonomic responses to lateralized cold pressor and facial cooling tasks. Psychophysiology.

[17] Wittling W. (1997). Brain asymmetry and autonomic control of the heart. Eur. Psychol..

[18] Andrew R.J. (1991). Neural and Behavioural Plasticity: The Use of the Domestic Chick as a Model.

[19] Andrew R.J. (1997). Left and right hemisphere memory traces: Their formation and fate. Evidence from events during memory formation in the chick. Later. Asymmetries Body Brain Cogn..

[20] Bisazza A., Cantalupo C., Capocchiano M., Vallortigara G. (2000). Population lateralisation and social behaviour: A study with 16 species of fish. Later. Asymmetries Body Brain Cogn..

[21] Rogers L.J. (2000). Evolution of hemispheric specialization: Advantages and disadvantages. Brain Lang..

[22] Vallortigara G. (2006). The evolutionary psychology of left and right: Costs and benefits of lateralization. Dev. Psychobiol..

[23] Zeder M.A., Gepts P., Famula T.R., Bettinger R.L., Brush S.B., Damania A.B., McGuire P.E., Qualset C.O. (2012). Pathways to animal domestication. Biodiversity in Agriculture: Domestication, Evolution, and Sustainability.

[24] Ghirlanda S., Frasnelli E., Vallortigara G. (2009). Intraspecific competition and coordination in the evolution of lateralization. Philos. Trans. R. Soc. Lond. B Biol. Sci..

[25] Frasnelli E., Vallortigara G. (2018). Individual-level and population-level lateralization: Two sides of the same coin. Symmetry.

[26] Darwin C. (1859). On the Origin of Species.

[27] Darwin C. (1868). The Variations of Plants and Animals under Domestication.

[28] Daisley J.N., Vallortigara G., Regolin L. (2010). Logic in an asymmetrical (social) brain: Transitive inference in the young domestic chick. Soc. Neurosci..

[29] Robins A., Goma A.A., Ouine L., Phillips C.J.C. (2018). The eyes have it: Lateralized coping strategies in cattle herds responding to human approach. Anim. Cogn..

[30] Rogers L.J., Andrew R. (2002). Comparative Vertebrate Lateralization.

[31] Farmer K., Krueger K., Byrne R.W. (2010). Visual laterality in the domestic horse (*Equus caballus*) interacting with humans. Anim. Cogn..

[32] Phillips C.J.C., Oevermans H., Syrett K.L., Jespersen A.Y., Pearce G.P. (2015). Lateralization of behavior in dairy cows in response to conspecifics and novel persons. J. Dairy Sci..

[33] Goma A.A., Pearce P.G., Uddin J., Rimon E., Davies H., Phillips C.J.C. (2018). A forced lateralisation test for dairy cows and its relation to their behaviour. Appl. Anim. Behav. Sci..

[34] Rizhova L.Y., Kokorina E.P. (2005). Behavioural asymmetry is involved in regulation of autonomic processes: Left side presentation of food improves reproduction and lactation in cows. Behav. Brain Res..

[35] Kappel S., Mendl M.T., Barrett D.C., Murrell J.C., Whay H.R. (2017). Lateralized behaviour as indicator of affective state in dairy cows. PLoS ONE.

[36] Austin N.P., Rogers L.J. (2012). Limb preferences and lateralization of aggression, reactivity and vigilance in feral horses, *Equus caballus*. Anim. Behav..

[37] Austin N.P., Rogers L.J. (2014). Lateralization of agonistic and vigilance responses in Przewalski horses (*Equus przewalskii*). Appl. Anim. Behav. Sci..

[38] Rogers L.J. (2010). Relevance of brain and behavioural lateralization to animal welfare. Appl. Anim. Behav. Sci..

[39] Haselton M.G., Nettle D. (2006). The paranoid optimist: An integrative evolutionary model of cognitive biases. Personal. Soc. Psychol. Rev..

[40] Van Vuure T. (2002). History, morphology and ecology of the Aurochs (*Bos primigenius*). Lutra.

[41] Zhang H., Paijmans J.L.A., Chang F., Wu X., Chen G., Lei C., Yang X., Wei Z., Bradley D.G., Orlando L. (2013). Morphological and genetic evidence for early Holocene cattle management in northeastern China. Nat. Commun..

[42] Pramod R.K., Velayutham D., Sajesh P.K., Beena P.S., Zachariah A., Zachariah A., Chandramohan B., Sujith S.S., Ganapathi P., Kumar B.D. (2019). Complete mitogenome reveals genetic divergence and phylogenetic relationships among Indian cattle (*Bos indicus*) breeds. Anim. Biotechnol..

[43] Ajmone-Marsan P., Garcia J.F., Lenstra J.A. (2010). On the origin of cattle: How aurochs became cattle and colonized the world. Evol. Anthropol. Issues News Rev..

[44] Beja-Pereira A., Caramelli D., Lalueza-Fox C., Vernesi C., Ferrand N., Casoli A., Goyache F., Royo L.J., Conti S., Lari M. (2006). The origin of European cattle: Evidence from modern and ancient DNA. Proc. Natl. Acad. Sci. USA.

[45] Götherström A., Anderung C., Hellborg L., Elburg R., Smith C., Bradley D.G., Ellegren H. (2005). Cattle domestication in the Near East was followed by hybridization with aurochs bulls in Europe. Proc. R. Soc. Lond. B Biol. Sci..

[46] Orlando L. (2015). The first aurochs genome reveals the breeding history of British and European cattle. Genome Biol..

[47] Upadhyay M.R., Chen W., Lenstra J.A., Goderie C.R.J., MacHugh D.E., Park S.D.E., Magee D.A., Matassino D., Ciani F., Megens H.-J. (2017). Genetic origin, admixture and population history of aurochs (*Bosprimigenius*) and primitive European cattle. Heredity.

[48] Wright E., Viner-Daniels S. (2015). Geographical variation in the size and shape of the European aurochs (*Bos primigenius*). J. Archaeol. Sci..

[49] Hristov P., Spassov N., Iliev N., Radoslavov G. (2017). An independent event of Neolithic cattle domestication on the South-eastern Balkans: Evidence from prehistoric aurochs and cattle populations. Mitochondrial DNA Part A.

[50] Meier J.S., Goring-Morris A.N., Munro N.D. (2017). Aurochs bone deposits at Kfar HaHoresh and the southern Levant across the agricultural transition. Antiquity.

[51] Goring-Morris A.N. (2005). Life, death and the emergence of differential status in the Near Eastern Neolithic: Evidence from Kfar HaHoresh, Lower Galilee, Israel. Archaeological Perspectives on the Transmission and Transformation of Culture in the Eastern Mediterranean.

[52] Twiss K.C., Russell N. (2009). Taking the bull by the horns: Ideology, masculinity, and cattle horns at Çatalhöyük (Turkey). Paléorient.

[53] Isaac E. (1962). On the domestication of cattle: Zoology and cultural history both illuminate the view that the original motive was religious, not economic. Science.

[54] Leeming D. (2005). The Oxford Companion to World Mythology.

[55] Smith M.S. (2001). The Origins of Biblical Monotheism: Israel’s Polytheistic Background and the Ugaritic Texts.

[56] Hart G. (2005). Routledge Dictionary of Egyptian Gods and Goddesses..

[57] Monaghan P. (2010). Goddesses in World Culture.

[58] Ellis N. (2010). Sekhmet, Bast, and Hathor: Power, passion, and transformation through the Egyptian goddess Trinity. Goddesses in World Culture.

[59] Daniels P.T. (1996). The first civilizations. The World’s Writing Systems.

[60] Daniels P.T. (2013). Scripts of semitic languages. The Semitic Languages.

[61] Daniels P.T. (1996). The World’s Writing Systems.

[62] Ritner R.K. (1996). Egyptian writing. The World’s Writing Systems.

[63] Robinson A. (2013). Writing systems. The Book: A Global History.

[64] Darnell J.C., Dobbs-Allsopp F.W., Lundberg M.J., McCarter P.K., Zuckerman B., Manassa C. (2005). Two early alphabetic inscriptions from the Wadi el-Ḥôl: New evidence for the origin of the alphabet from the Western Desert of Egypt. Annu. Am. Sch. Orient. Res..

[65] Darnell J.C. (2013). Wadi el-Hol. UCLA Encycl. Egyptol..

[66] Colles B.E. (2010). Proto–Alphabetic inscriptions from the Wadi Arabah. Antig. Oriente.

[67] Simons F. (2011). Proto-Sinaitic–progenitor of the alphabet. Rosetta.

[68] Kaufman S.A. (1986). The pitfalls of typology: On the early history of the alphabet. Hebr. Union Coll. Annu..

[69] Millard A.R. (1986). The infancy of the alphabet. World Archaeol..

[70] Coogan M.D. (1974). Alphabets and elements. Bull. Am. Sch. Orient. Res..

[71] Stieglitz R.R. (1971). The Ugaritic cuneiform and Canaanite linear alphabets. J. East. Stud..

[72] O’Connor M. (1996). Epigraphic Semitic scripts. The World’s Writing Systems.

[73] Naveh J. (1973). Some semitic epigraphical considerations on the antiquity of the Greek alphabet. Am. J. Archaeol..

[74] Threatte L. (1996). The Greek alphabet. The World’s Writing Systems.

[75] Luraghi N. (2010). The local scripts from nature to culture. Class. Antiq..

[76] Chrisomalis S. (2003). The Egyptian origin of the Greek alphabetic numerals. Antiq. Camb..

[77] Pande N.A. (2010). Numeral systems of great ancient human civilizations. J. Sci. Arts.

[78] Cauvin J. (2002). The symbolic foundations of the Neolithic Revolution in the Near East. Life in Neolithic Farming Communities.

[79] Ibáñez J.J., González-Urquijo J., Teira-Mayolini L.C., Lazuén T. (2018). The emergence of the Neolithic in the Near East: A protracted and multi-regional model. Quat. Int..

[80] Zeder M.A. (2009). The neolithic macro-(R)evolution: Macroevolutionary theory and the study of culture change. J. Archaeol. Res..

[81] Zeder M.A., Prentiss A., Kuijt I., Chatters J.C. (2009). Evolutionary biology and the emergence of agriculture: The value of co-opted models of evolution in the study of culture change. Macroevolution in Human Prehistory: Evolutionary Theory and Processual Archaeology.

[82] Cross F.M. (2009). Canaanite Myth and Hebrew Epic: Essays in the History of the Religion of Israel.

[83] Benner J.A. (2005). The Ancient Hebrew lexicon of the Bible.

[84] Siniscalchi M., D’Ingeo S., Quaranta A. (2017). Lateralized functions in the dog brain. Symmetry.

[85] Fabre-Thorpe M., Fagot J., Lorincz E., Levesque F., Vauclair J. (1993). Laterality in cats: Paw preference and performance in a visuomotor activity. Cortex.

[86] Wells D.L., Millsopp S. (2009). Lateralized behaviour in the domestic cat, Felis silvestris catus. Anim. Behav..

[87] McDowell L.J., Wells D.L., Hepper P.G. (2018). Lateralization of spontaneous behaviours in the domestic cat, *Felis silvestris*. Anim. Behav..

[88] Camerlink I., Menneson S., Turner S.P., Farish M., Arnott G. (2018). Lateralization influences contest behaviour in domestic pigs. Sci. Rep..

[89] Espmark Y., Kinderås K. (2002). Behavioural lateralisation in reindeer. Rangifer.

[90] Anderson D.M., Murray L.W. (2013). Sheep laterality. Laterality Asymmetries Body Brain Cogn..

[91] Barnard S., Matthews L., Messori S., Podaliri-Vulpiani M., Ferri N. (2016). Laterality as an indicator of emotional stress in ewes and lambs during a separation test. Anim. Cogn..

[92] Versace E., Morgante M., Pulina G., Vallortigara G. (2007). Behavioural lateralization in sheep (*Ovis aries*). Behav. Brain Res..

[93] Lane A., Phillips C. (2004). A note on behavioural laterality in neonatal lambs. Appl. Anim. Behav. Sci..

[94] Nawroth C., Albuquerque N., Savalli C., Single M.-S., McElligott A.G. (2018). Goats prefer positive human emotional facial expressions. R. Soc. Open Sci..

[95] Austin N.P., Rogers P.L.J. (2007). Asymmetry of flight and escape turning responses in horses. Lateral. Asymmetries Body Brain Cogn..

[96] Smith A.V., Proops L., Grounds K., Wathan J., Scott S.K., McComb K. (2018). Domestic horses (*Equus caballus*) discriminate between negative and positive human nonverbal vocalisations. Sci. Rep..

[97] Hamp E. (2001). A rule of the road. Gen. Linguist. Univ. Park Pa.

[98] Phillips C. (2008). Cattle Behaviour and Welfare.

[99] Sullivan R.M., Gratton A. (1999). Lateralized effects of medial prefrontal cortex lesions on neuroendocrine and autonomic stress responses in rats. J. Neurosci..

[100] Wingfield J.C. (2005). The concept of allostasis: Coping with a capricious environment. J. Mammal..

[101] Evans C.S., Evans L., Marler P. (1993). On the meaning of alarm calls: Functional reference in an avian vocal system. Anim. Behav..

[102] Dharmaretnam M., Rogers L.J. (2005). Hemispheric specialization and dual processing in strongly versus weakly lateralized chicks. Behav. Brain Res..

[103] Revell D., Maynard B., Erkelenz P., Thomas D. ‘Rangeland Self Herding’-positively influencing grazing distribution to benefit livestock, landscapes and people. Proceedings of the 18th Australian Rangeland Society Biennial Conference, Australian Rangeland Society.

[104] Cerqueira J.J., Almeida O.F.X., Sousa N. (2008). The stressed prefrontal cortex. Left? Right!. Brain. Behav. Immun..

[105] Meador K.J., Loring D.W., Ray P.G., Helman S.W., Vazquez B.R., Neveu P.J. (2004). Role of cerebral lateralization in control of immune processes in humans. Ann. Neurol..

[106] Neveu P.J. (2002). Cerebral lateralization and the immune system. Int. Rev. Neurobiol..

[107] Stoyanov Z., Decheva L., Pashalieva I., Nikolova P. (2012). Brain asymmetry, immunity, handedness. Open Med..

[108] Sumner R.C., Parton A., Nowicky A.V., Kishore U., Gidron Y. (2011). Hemispheric lateralisation and immune function: A systematic review of human research. J. Neuroimmunol..

